# Supplementary material for: Content-rich biological network constructed by mining PubMed abstracts
Source: BMC Bioinformatics. 2004 Oct 8;5:147. doi: 10.1186/1471-2105-5-147 (PMC528731; doi:10.1186/1471-2105-5-147)
Supplement: Additional File 2 — The original results of the above study (non-essential files are deleted to keep the file size under the limit set by BMC bioinformatics). [file 1471-2105-5-147-S2.bz2 › chilibotAdditionalFile2/dip05/656ID10449718E11047/html/TRPE_TRPG.html]

 


 **TRPE** and **TRPG** 
  
Found 35 abstracts in PubMed, retrieved 05.  
 

 What does Google say? 
 PDF only 
| .edu only 

---

**Interactive relationship** (e.g. stimulation, inhibition, etc)

**Non-interactive relationship** (e.g. studied together, co-existance, homology, etc.)

- Tk  **trpE**  and Tk  **trpG** , the genes that encode the two subunits of anthranilate synthase from the hyperthermophilic archaeon Thermococcus kodakaraensis KOD1, have been expressed independently in Escherichia coli.  Ref: 11237738 Biochem Biophys Res Commun, 2001
- In bacteria, the first reaction of the tryptophan biosynthetic pathway involves the conversion of chorismate and glutamine to anthranilate by the action of anthranilate synthase, which is composed of the alpha  **trpE**  gene product and beta  **trpG**  gene product subunits.  Ref: 12634337 Microbiology, 2003
- The anthranilate synthase complex Tk AS complex was obtained by heat treatment of the mixture of cell free extracts containing each recombinant protein, Tk  **TrpE**  alpha subunit and Tk  **TrpG**  beta subunit, at 85 degrees C for 10 min.  Ref: 11237738 Biochem Biophys Res Commun, 2001
- Nucleotide sequences upstream of  **trpE**  and  **trpG**  encode putative leader peptides similar in sequence to leader peptides found in other bacterial species, and the phenotypes of the mutants strongly suggest that transcription of these operons is regulated solely by attenuation.  Ref: 11325956 J Bacteriol, 2001
- A systematic polymerase chain reaction PCR analysis of P. aeruginosa strains that produce various subtypes of R and F pyocins revealed that the genes for every subtype are located between  **trpE**  and  **trpG**  in the same or very similar gene organization as for R2 and F2 pyocins, but with alterations in genes that determine the receptor specificity.  Ref: 11069649 Mol Microbiol, 2000
